# Supplementary material for: ULTRA-effective labeling of tandem repeats in genomic sequence
Source: Bioinform Adv. 2024 Oct 9;4(1):vbae149. doi: 10.1093/bioadv/vbae149 (PMC11580682; doi:10.1093/bioadv/vbae149)
Supplement: vbae149_Supplementary_Data [file vbae149_supplementary_data.pdf]

Supplementary Material for ULTRA-Effective Labeling of Tandem Repeats in Genomic Sequence

Daniel R. Olson and Travis J. Wheeler

September 20, 2024

1 Coverage without minimum repeat number

These experiments were performed as described in Section 3.1 of the main paper, although without the constraint of needing a minimum number of repeat units to annotate a tandem repeat; to that end *tantan* was run with the flag `-f3` instead of `-f4`, and ULTRA was run with `--min_unit 0`.

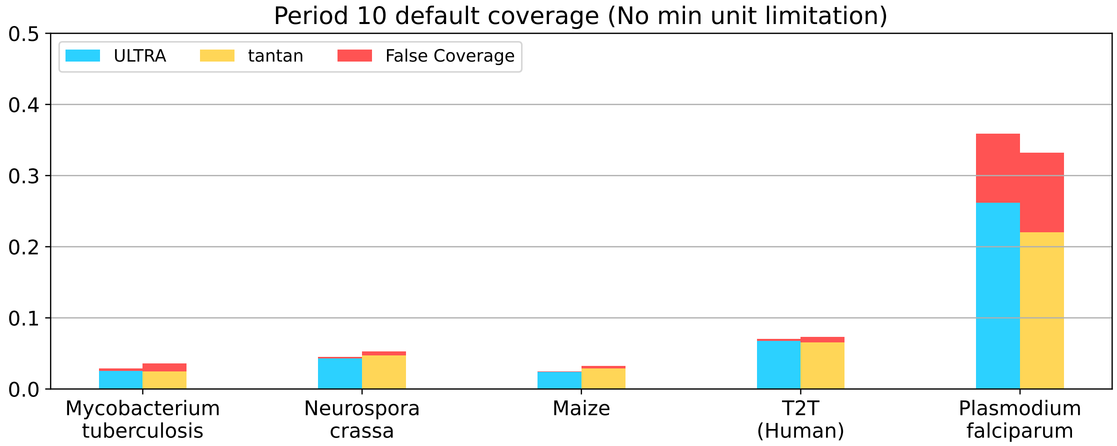

Supplementary Figure 1. Period 10 coverage with default settings (no minimum repeat unit limit)

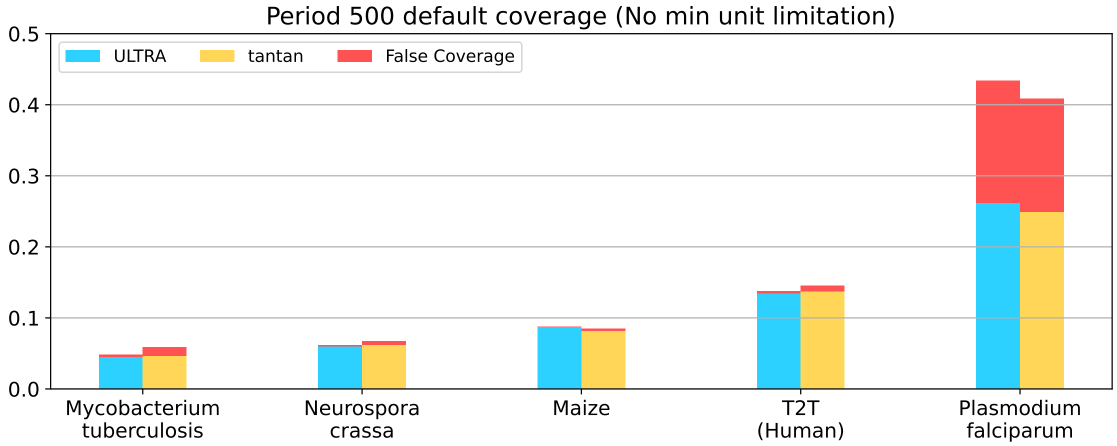

Supplementary Figure 2. Period 500 coverage with default settings (no minimum repeat unit limit)

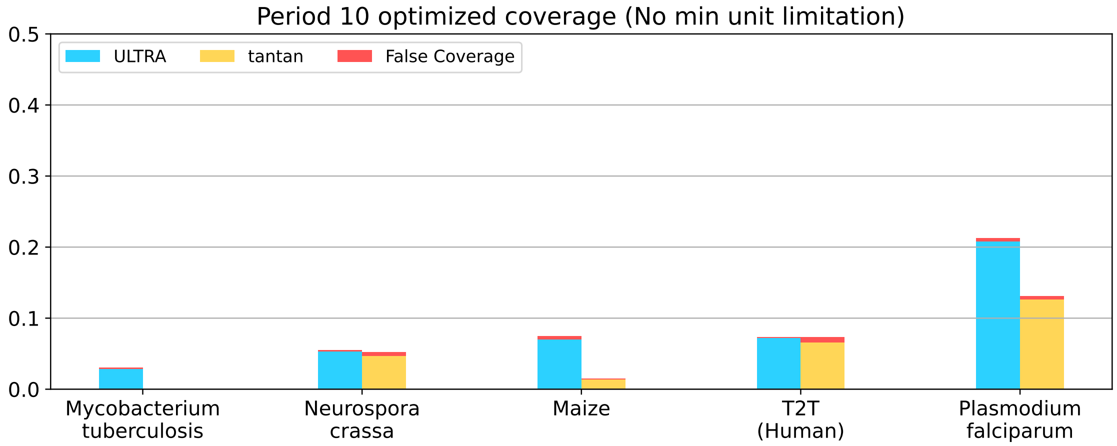

Supplementary Figure 3. Period 10 coverage with optimized settings (no minimum repeat unit limit)

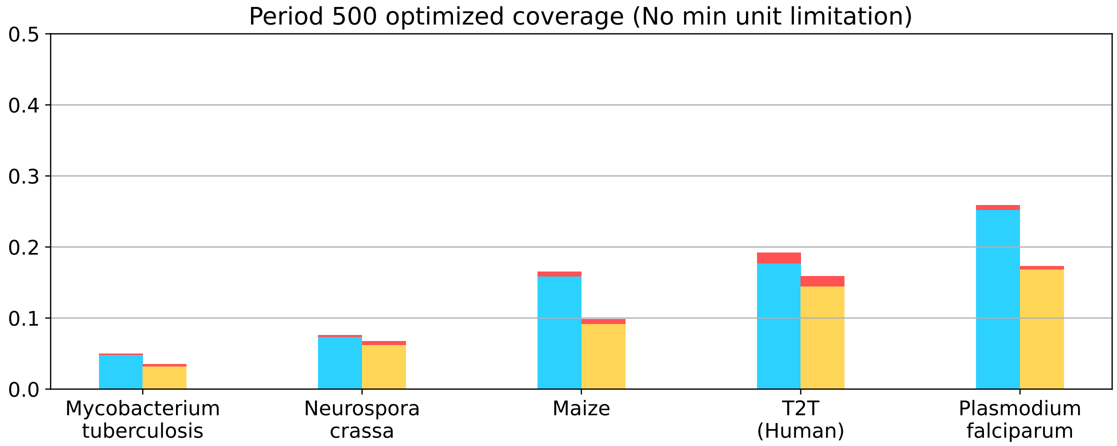

Supplementary Figure 4. Period 500 coverage with optimized settings (no minimum repeat unit limit)

2 Coverage tables and optimized parameters

The tables in this section contain numeric results for all coverage experiments. Tables corresponding to grid-optimized coverage also contain the parameters used. The placeholders [at matrix] and [gc matrix] found in some *tantan* parameters refer to a file path for an at-rich and gc-rich *tantan* scoring matrix respectively. The at-rich scoring matrix is distributed with the *tantan* source code (`tantan/test/atMask.mat`). The gc-rich scoring matrix is identical to the at-rich scoring matrix, except for the scores of `AA` and `TT`, which are swapped with the scores for `CC` and `GG`.

Supplementary Table 1. Period 10 coverage (default settings)

Genome	Coverage	Shuffled Coverage
**ULTRA**		
Mycobacterium tuberculosis	2.85	0.30
Neurospora crassa	4.45	0.14
Maize	2.44	0.06
T2T	7.00	0.21
Plasmodium falciparum	35.88	9.67
**ULTRA --tune**		
Mycobacterium tuberculosis	3.87	0.22
Neurospora crassa	13.20	1.22
Maize	13.20	1.38
T2T	15.83	1.61
Plasmodium falciparum	43.83	8.21
**tantan**		
Mycobacterium tuberculosis	0.88	0.29
Neurospora crassa	2.30	0.14
Maize	1.08	0.10
T2T	3.57	0.19
Plasmodium falciparum	16.22	2.00
**TRF**		
Mycobacterium tuberculosis	1.18	0.20
Neurospora crassa	2.00	0.12
Maize	0.99	0.09
T2T	4.41	0.15
Plasmodium falciparum	15.85	1.76

Supplementary Table 2. Period 500 coverage (default settings)

**Genome**	**Coverage**	**Shuffled Coverage**
	**ULTRA**	
Mycobacterium tuberculosis	4.70%	0.33%
Neurospora crassa	5.95%	0.18%
Maize	7.61%	0.06%
T2T	12.14%	0.28%
Plasmodium falciparum	43.34%	17.19%
	**ULTRA --tune**	
Mycobacterium tuberculosis	6.63%	0.34%
Neurospora crassa	19.79%	2.98%
Maize	25.65%	3.35%
T2T	29.40%	6.43%
Plasmodium falciparum	41.85%	5.89%
	**tantan**	
Mycobacterium tuberculosis	1.10%	0.19%
Neurospora crassa	2.33%	0.09%
Maize	3.60%	0.07%
T2T	6.50%	0.13%
Plasmodium falciparum	18.55%	1.64%
	**TRF**	
Mycobacterium tuberculosis	-	-
Neurospora crassa	3.00%	0.18%
Maize	8.20%	0.45%
T2T	12.89%	0.82%
Plasmodium falciparum	-	-

Supplementary Table 3. Period 10 coverage (optimized settings)

Genome	Coverage	Shuffled Coverage	Arguments
		**ULTRA**	
Mycobacterium tuberculosis	3.07%	0.18%	-p 10 -m 0.6 --at 0.5
Neurospora crassa	5.51%	0.17%	-p 10 -m 0.6 --at 0.6
Maize	7.48%	0.45%	-p 10 -m 0.6 --at 0.4
T2T	7.37%	0.14%	-p 10 -m 0.6 --at 0.5
Plasmodium falciparum	21.25%	0.49%	-p 10 -m 0.7 --at 0.6
		**tantan**	
Mycobacterium tuberculosis	-	-	-
Neurospora crassa	2.60%	0.20%	-w 10 -f 3 -r 0.005 -e 0.05
Maize	1.08%	0.11%	-w 10 -f 3 -r 0.005 -e 0.05 -s 0.85
T2T	3.82%	0.27%	-w 10 -f 3 -r 0.01 -e 0.05
Plasmodium falciparum	12.55%	0.08%	-w 10 -f 3 -r 0.01 -e 0.05 -s 0.85 -m [at matrix]
		**TRF**	
Mycobacterium tuberculosis	-	-	-
Neurospora crassa	3.45%	0.30%	2 5 5 80 10 30 10 -l 12 -h -ngs
Maize	0.99%	0.09%	2 7 7 80 10 30 10 -l 12 -h -ngs
T2T	5.09%	0.40%	2 5 5 80 10 30 10 -l 12 -h -ngs
Plasmodium falciparum	-	-	-

Supplementary Table 4. Period 500 coverage (optimized settings)

Genome	Coverage	Shuffled Coverage	Arguments
		**ULTRA**	
Mycobacterium tuberculosis	4.92%	0.20%	-p 500 -m 0.6 --at 0.5
Neurospora crassa	7.47%	0.22%	-p 500 -m 0.6 --at 0.6
Maize	15.95%	0.65%	-p 500 -m 0.6 --at 0.4
T2T	15.37%	1.48%	-p 500 -m 0.7 --at 0.7
Plasmodium falciparum	41.85%	5.89%	-p 500 -m 0.7 --at 0.6
		**tantan**	
Mycobacterium tuberculosis	1.60%	0.12%	-w 500 -f 3 -r 0.005 -e 0.05 -m [gc matrix]
Neurospora crassa	2.88%	0.20%	-w 500 -f 3 -r 0.01 -e 0.05 -s 0.85
Maize	3.97%	0.15%	-w 500 -f 3 -r 0.01 -e 0.05
T2T	11.35%	0.82%	-w 500 -f 3 -r 0.005 -e 0.05 -s 0.85 -m [at matrix]
Plasmodium falciparum	18.55%	1.64%	-w 500 -f 3 -r 0.005 -e 0.05
		**TRF**	
Mycobacterium tuberculosis	-	-	-
Neurospora crassa	3.00%	0.18%	2 7 7 80 10 30 500 -l 12 -h -ngs
Maize	8.20%	0.45%	2 5 5 80 10 30 500 -l 12 -h -ngs
T2T	12.89%	0.82%	2 5 5 80 10 30 500 -l 12 -h -ngs
Plasmodium falciparum	-	-	-

Supplementary Table 5. Period 10 coverage (default settings, no minimum unit)

**Genome**	**Coverage**	**Shuffled Coverage**	**Arguments**
	**ULTRA**		
Mycobacterium tuberculosis	2.85%	0.30%	-p 10 --min_unit 0
Neurospora crassa	4.45%	0.14%	-p 10 --min_unit 0
Maize	2.44%	0.06%	-p 10 --min_unit 0
T2T	7.00%	0.21%	-p 10 --min_unit 0
Plasmodium falciparum	35.88%	9.67%	-p 10 --min_unit 0
	**tantan**		
Mycobacterium tuberculosis	3.56%	1.08%	-w 10 -f 3
Neurospora crassa	5.24%	0.51%	-w 10 -f 3
Maize	3.22%	0.33%	-w 10 -f 3
T2T	7.32%	0.73%	-w 10 -f 3
Plasmodium falciparum	33.21%	11.12%	-w 10 -f 3

Supplementary Table 6. Period 500 coverage (default settings, no minimum unit)

**Genome**	**Coverage**	**Shuffled Coverage**	**Arguments**
	**ULTRA**		
Mycobacterium tuberculosis	4.87%	0.33%	-p 500 --min_unit 0
Neurospora crassa	6.15%	0.18%	-p 500 --min_unit 0
Maize	8.82%	0.06%	-p 500 --min_unit 0
T2T	13.73%	0.28%	-p 500 --min_unit 0
Plasmodium falciparum	43.44%	17.23%	-p 500 --min_unit 0
	**tantan**		
Mycobacterium tuberculosis	5.86%	1.20%	-w 500 -f 3
Neurospora crassa	6.74%	0.56%	-w 500 -f 3
Maize	8.51%	0.35%	-w 500 -f 3
T2T	14.55%	0.82%	-w 500 -f 3
Plasmodium falciparum	40.82%	15.92%	-w 500 -f 3

Supplementary Table 7. Period 10 coverage (optimized settings, no minimum unit)

Genome	Coverage	Shuffled Coverage	Arguments
		**ULTRA**	
Mycobacterium tuberculosis	3.07%	0.18%	--min_unit 0 -p 10 -m 0.6 --at 0.5
Neurospora crassa	5.51%	0.17%	--min_unit 0 -p 10 -m 0.6 --at 0.6
Maize	7.48%	0.45%	--min_unit 0 -p 10 -m 0.6 --at 0.5
T2T	7.37%	0.14%	--min_unit 0 -p 10 -m 0.6 --at 0.5
Plasmodium falciparum	21.25%	0.49%	--min_unit 0 -p 10 -m 0.7 --at 0.5
		**tantan**	
Mycobacterium tuberculosis	-	-	-
Neurospora crassa	5.24%	0.51%	-w 10 -f 3 -r 0.005 -e 0.05
Maize	1.50%	0.08%	-w 10 -f 3 -r 0.01 -e 0.05 -s -0.85
T2T	7.32%	0.73%	-w 10 -f 3 -r 0.005 -e 0.05
Plasmodium falciparum	13.09%	0.44%	-w 10 -f 3 -r 0.01 -e 0.05 -m [at matrix]

Supplementary Table 8. Period 500 coverage (optimized settings, no minimum unit)

Genome	Coverage	Shuffled Coverage	Arguments
		**ULTRA**	
Mycobacterium tuberculosis	4.98%	0.20%	--min_unit 0 -p 500 -m 0.6 --at 0.5
Neurospora crassa	7.58%	0.22%	--min_unit 0 -p 500 -m 0.6 --at 0.6
Maize	16.53%	0.65%	--min_unit 0 -p 500 -m 0.6 --at 0.4
T2T	19.21%	1.50%	--min_unit 0 -p 500 -m 0.7 --at 0.7
Plasmodium falciparum	25.88%	0.66%	--min_unit 0 -p 500 -m 0.7 --at 0.6
		**tantan**	
Mycobacterium tuberculosis	3.54%	0.35%	-w 500 -f 3 -r 0.01 -e 0.05 -s -0.85
Neurospora crassa	6.74%	0.56%	-w 500 -f 3 -r 0.005 -e 0.05
Maize	9.88%	0.69%	-w 500 -f 3 -r 0.01 -e 0.05
T2T	15.91%	1.44%	-w 500 -f 3 -r 0.01 -e 0.05
Plasmodium falciparum	17.31%	0.47%	-w 500 -f 3 -r 0.01 -e 0.05 -m [at matrix]
